# A Pediatric Knee Exoskeleton With Real-Time Adaptive Control for Overground Walking in Ambulatory Individuals With Cerebral Palsy

**DOI:** 10.3389/frobt.2021.702137

**Published:** 2021-06-18

**Authors:** Ji Chen, Jon Hochstein, Christina Kim, Luke Tucker, Lauren E. Hammel, Diane L. Damiano, Thomas C. Bulea

**Affiliations:** ^1^ Biomedical Engineering Program, Department of Mechanical Engineering, University of the District of Columbia, Washington, DC, United States; ^2^ Rehabilitation Medicine Department, National Institutes of Health Clinical Center, Bethesda, MD, United States

**Keywords:** pediatric knee exoskeleton, real-time adaptive control, crouch gait, finite state machine, torque assistance, cerebral palsy

## Abstract

Gait training *via* a wearable device in children with cerebral palsy (CP) offers the potential to increase therapy dosage and intensity compared to current approaches. Here, we report the design and characterization of a pediatric knee exoskeleton (P.REX) with a microcontroller based multi-layered closed loop control system to provide individualized control capability. Exoskeleton performance was evaluated through benchtop and human subject testing. Step response tests show the averaged 90% rise was 26 ± 0.2 ms for 5 Nm, 22 ± 0.2 ms for 10 Nm, 32 ± 0.4 ms for 15 Nm. Torque bandwidth of P.REX was 12 Hz and output impedance was less than 1.8 Nm with control on (Zero mode). Three different control strategies can be deployed to apply assistance to knee extension: state-based assistance, impedance-based trajectory tracking, and real-time adaptive control. One participant with typical development (TD) and one participant with crouch gait from CP were recruited to evaluate P.REX in overground walking tests. Data from the participant with TD were used to validate control system performance. Kinematic and kinetic data were collected by motion capture and compared to exoskeleton on-board sensors to evaluate control system performance with results demonstrating that the control system functioned as intended. The data from the participant with CP are part of a larger ongoing study. Results for this participant compare walking with P.REX in two control modes: a state-based approach that provided constant knee extension assistance during early stance, mid-stance and late swing (Est+Mst+Lsw mode) and an Adaptive mode providing knee extension assistance proportional to estimated knee moment during stance. Both were well tolerated and significantly improved knee extension compared to walking without extension assistance (Zero mode). There was less reduction in gait speed during use of the adaptive controller, suggesting that it may be more intuitive than state-based constant assistance for this individual. Future work will investigate the effects of exoskeleton assistance during overground gait training in children with neurological disorders and will aim to identify the optimal individualized control strategy for exoskeleton prescription.

## Introduction

Effective gait rehabilitation remains a significant challenge in children with cerebral palsy (CP) (Damiano and DeJong, 2009). One major hurdle is achieving the required dosage and intensity of gait training necessary to produce meaningful long term improvements in walking ability. Therapist-assisted training is resource-intensive and therefore limited to several sessions per week, each lasting less than an hour. Robotic assisted gait trainers provide more efficient delivery of traditional assistance-based therapy which was postulated to enhance therapeutic benefits due to repetitive task specific practice, which could facilitate muscle strengthening and motor improvement. Robotic trainers have shown some effectiveness in CP (Dodd and Foley, 2007) but results from controlled clinical trials indicate that device assisted approaches are not superior to traditional therapy of equal intensity (Dobkin and Duncan, 2012).

One drawback of robotic treadmill-based approaches is that they are limited to clinical and/or research environments, which constrain the treatment frequency, duration and intensity making it difficult to translate improvements to overground walking after training (Dobkin and Duncan, 2012). A further drawback of robotic assisted gait trainers, particularly in an already ambulatory population such as children with gait pathology from CP, is the way in which the robot interacts with the user in these systems. Specifically, most treadmill based robotic systems use an approach whereby the robotic assistance attempts to guide the limbs toward a pre-defined target trajectory (Veneman et al., 2007). In practice, this approach is implemented as an impedance-based assist-as-needed paradigm such that robotic assistance is only provided if the user deviates from the target trajectory (Marchal-Crespo and Reinkensmeyer, 2009). Rationale for this assistive approach centers on the notion that repetitive task training will induce plasticity and ultimately improve function, in response to experiencing somatosensory input that might not otherwise be possible (Harkema, 2001; Marchal-Crespo and Reinkensmeyer, 2009). While this framework is plausible, though not yet proven, for individuals who lack the volitional control to walk on their own, its applicability to rehabilitation of ambulatory individuals is not clear. In children and adults with gait pathology and capacity for motor learning, the goal is different than in those with paralysis. Rather than replace lost or absent function, gait training aims to improve the participant’s baseline walking pattern by encouraging longer bouts of training and exercise. This goal may be combined in some more affected individuals with the secondary objective to make walking easier and/or safer.

Wearable robotic exoskeletons have the potential to advance the field of locomotor training by enabling practice to occur outside the clinic environment, which could greatly increase the amount and intensity of training. There are now several commercially available exoskeletons, such as the EKSO, Indego, ReWalk, and ExoAtlet, which are primarily designed to restore walking function to individuals with paralysis from spinal cord injury (SCI) (Farris et al., 2014; Onose et al., 2016) or stroke (Bortole et al., 2015) and mirror the assist-as-needed approach of treadmill-based robotics. Because of the additional requirement of providing considerable anti-gravity support, these wearable devices are cumbersome and crutches or other walking aids may also be needed to provide stability for over-ground walking. Several robotic exoskeletons have recently been developed specifically for pediatric populations to assist gait (Lerner et al., 2016a; Bayón et al., 2017; Patané et al., 2017; Cestari et al., 2018; Lerner et al., 2018). For example, the EXOTrainer (Cestari et al., 2018) and WAKE-Up (Patané et al., 2017) use series elastic actuation (SEA) to enhance joint compliance in human and robot interactions as the elastic element helps reduce the interface stiffness and mechanical impedance, and increase the capacity for energy storage, all of which can aid in the use of wearable robots for rehabilitation of individuals who still have some voluntary movement (Kong et al., 2012). However, SEAs and other mechanisms designed to reduce interaction forces on the user (Zhou et al., 2018) increase system complexity and reduce dynamic responsiveness and may therefore limit their utility in children who are ambulatory and require relatively low levels of assistance at specific times during walking. Furthermore, these exoskeletons are primarily laboratory-based and not yet available for home and community-based gait training. In addition to recent advances in hardware, a variety of control strategies have recently been reported for use in ambulatory individuals that aim to provide adaptive or real-time adjustable assistance to the knee and/or ankle joint during walking. For example, an adaptive robust integral of sign error (or adaptive RISE) controller has been developed and implemented for a knee exoskeleton that achieves excellent trajectory tracking while also providing asymptotic stability (Sherwani et al., 2020). A feature-based knee moment estimation algorithm was developed for the NIH pediatric exoskeleton to provide real-time assistance that is proportional to user effort (Lerner et al., 2016b). At the ankle, a human-in-loop assistance strategy was developed by using co-adaptive control based on measured muscle activity and joint kinematics to continuously respond to user needs, resulting in assistance profiles that reduced the metabolic costs of walking in healthy individuals (Jackson and Collins, 2019). Additionally, a proportional joint moment control strategy was developed for an ankle exoskeleton in children with CP to provide assistance as a function of the instantaneous estimate of the ankle joint moment based on an in-sole foot sensor, resulting in significant reduction in the metabolic cost of transport (Gasparri et al., 2019).

Clearly, if the goal is to train a new pattern of walking, maintaining or increasing volitional muscle activity during exoskeleton use is critical from a motor learning standpoint (Lotze et al., 2003). We previously reported a novel pediatric exoskeleton prototype developed for children with crouch gait, or persistent knee flexion, due to CP (Lerner et al., 2016a). Rather than guiding the limbs toward a target trajectory, our strategy provided precisely timed knee extension assistance during stance and late swing phases to dynamically change posture. At the end of stance phase, the assistance was turned off requiring the user to adapt to the new position using their volitional muscle activity. An observational cohort study showed that this approach was safe and well tolerated; children as young as 5 years of age quickly acclimated to walking overground with the exoskeleton. More importantly, knee extension was significantly increased and knee extensor muscle activity was maintained during overground walking with the exoskeleton (Lerner et al., 2017a), demonstrating that a new gait pattern could be enabled and integrated with the user's own voluntary control capabilities.

Based on these initial studies (Lerner et al., 2016a; Lerner et al., 2017a; Lerner et al., 2017b), a second prototype of pediatric knee exoskeleton (P.REX) was designed to expand the population of users, and to enable its safe and effective use outside of the laboratory environment. For example, the effect of knee extension assistance on biomechanics and muscle activity during walking in children with crouch gait varied across participants (Lerner et al., 2017a; Lerner et al., 2017b) suggesting that additional improvements may be achieved by incorporation of control strategies which can be tailored to the individual. Thus, beyond refinement of the mechanical design, we introduce control strategies that can adjust assistance based on interaction level between the user and the device to facilitate motor learning for long-term skill retention (Joiner and Smith, 2008). The ultimate goal was to design a pediatric exoskeleton that is less encumbering and more powerful with the ability of adaptive control, and to evaluate its performance in bench top and human subject testing.

## Methods

### Mechatronic Design

**FIGURE 1:**
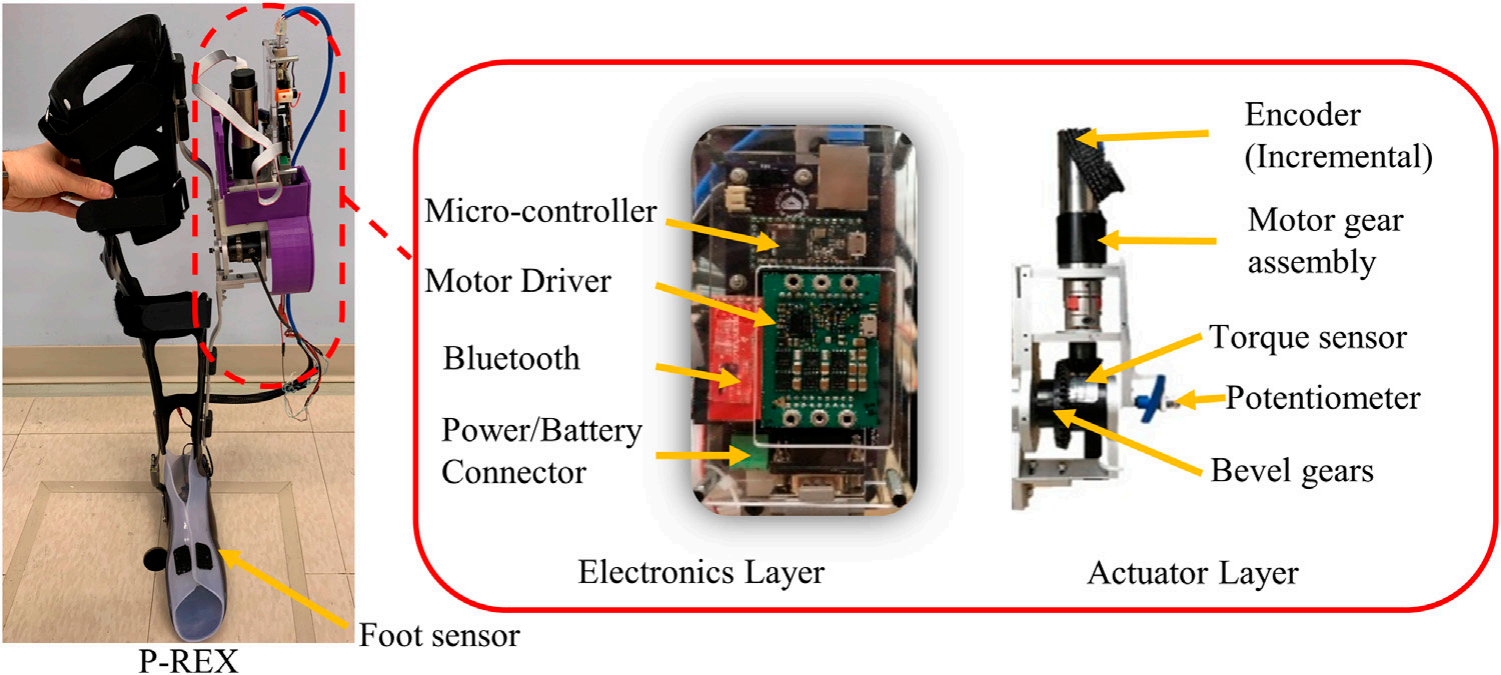
Overview of the pediatric knee exoskeleton (P.REX). The device incorporates a custom actuation assembly and embedded electronic control system mounted on the lateral side of the leg.

The mechatronic design of the exoskeleton used in this study has been previously reported (Chen et al., 2018) and the details are briefly summarized here. The mechatronic design aimed to provide maximum continuous assistance torque of 15 Nm at the velocity of 360°/sec. This design goal was based on our previous study that recorded peak swing knee extension velocity of 350°/sec (Damiano et al., 2006) in some children. A previous study (Patané et al., 2017) also reported that the maximum torque exerted at the knee by typically developing children during walking is 1 Nm/kg. We specified that our actuator should have the ability to provide at maximum 50% assistance of maximum knee joint moment for a child of approximately 30 kg. A right angle transmission was incorporated to allow actuator mounting lateral to the thigh (Figure 1) to ease donning and doffing and reduce possible restriction of the knee extension in the plane of progression. A modular design was utilized for easy assembly. A customized bevel gear set with a 3:1 reduction ratio was used with a Maxon motor and planetary gearhead GP 32C (51:1 reduction ratio) to meet torque and speed assistance specifications. In addition to high efficiency, the gear transmission provided easier assembly, but at the cost of slightly increased mass. To minimize weight, the bevel gear was machined to provide only the specified knee flexion/extension range of motion (120°). An inline reaction torque sensor was mounted on the knee shaft between the gear and lower leg attachment as shown in Figure 1. This sensor placement was based on the design used by Vischer and Khatib (Vischer and Khatib, 1990). The exoskeleton was designed to deliver a maximum continuous assistive torque of 15.7 Nm (at ∼8 A current) at a joint velocity 360°/sec. A MR series encoder with the capacity of 500 counts per turn was used to meet the specified resolution for angular position 0.1° and speed 0.1°/sec.

The motor assembly unit was connected to the lower (shank) and upper (thigh) uprights by custom designed brackets. Each upright connects to an orthotic brace (Ultraflex, Pottstown, PA) (Figure 1) *via* a simple latch mechanism to allow quick assembly/disassembly (Lerner et al., 2016a). Mechanical stops limit knee flexion to 105° and hyperextension to 10°. The molded brace is secured to the subject’s leg with Velcro straps. A challenge to applying knee torques is avoiding discomfort from the large reactionary sagittal plane forces on the leg. To reduce these forces, the thermoplastic shells are designed to cover a large area of soft tissue.

The first generation tethered control hardware (Lerner et al., 2016a) was replaced by embedded electronics with wireless communication to allow use in more naturalistic settings. The electronic enclosure containing a custom designed control circuit board was placed lateral to limb above the actuator (Figure 1). The electronic system provides power and signal conditioning for sensors and feedback control of the motor. The commercial servo controller enabled closed loop current (torque) control of the exoskeleton motor and knee joint angle measurement based on quadrature encoder reading. Knee angle and angular velocity, force sensitive resistor (FSR) (Interlink Electronics, Westlake Village, CA), and joint torque signals are inputs for a feedback control system implemented in an ARM microcontroller for real-time control (Teensy 3.2, PJRC.com, LLC, Sherwood, OR) (Figure 1). Reference knee angle for the encoder is set using a rotary potentiometer mounted on the actuator shaft. Full knee extension was set to 0°. Knee angle increases positively as it flexes. FSRs were mounted on shoe insoles to provide information regarding foot to ground contact. The FSR voltage output varied from 0 to 3.3 V. This reading was used to determine the state of gait as either stance or swing (Lerner et al., 2016a). An emergency switch, which can be carried by the user or a physical therapist, can be pressed to immediately cut off power from the system if necessary. The exoskeleton can be tethered to a DC power source in the laboratory setting or powered by a 24 V lithium-ion battery for out of lab use. The mass properties of the exoskeleton are provided in Table 1. The overall mechatronic design used a modular design concept which allows the actuator and electronic hardware to be easily attached to any knee ankle foot orthosis (KAFO) shells *via* metal uprights.

**TABLE 1:** Mass properties of the exoskeleton.

Segment	Weight^a^ (kg)	Ixx (kgm^2^)	Iyy (kgm^2^)	Izz (kgm^2^)
Thigh	1.77	0.268	0.296	0.031
Shank	0.62	0.039	0.040	0.003
Foot	0.20	0.002	0.001	0.001

a Total exoskeleton weight per limb 2.59 kg which includes embedded electronics. The axes are defined as follows: x anterior-posterior; y: superior-inferior; z: medial-lateral.

### Control

**FIGURE 2:**
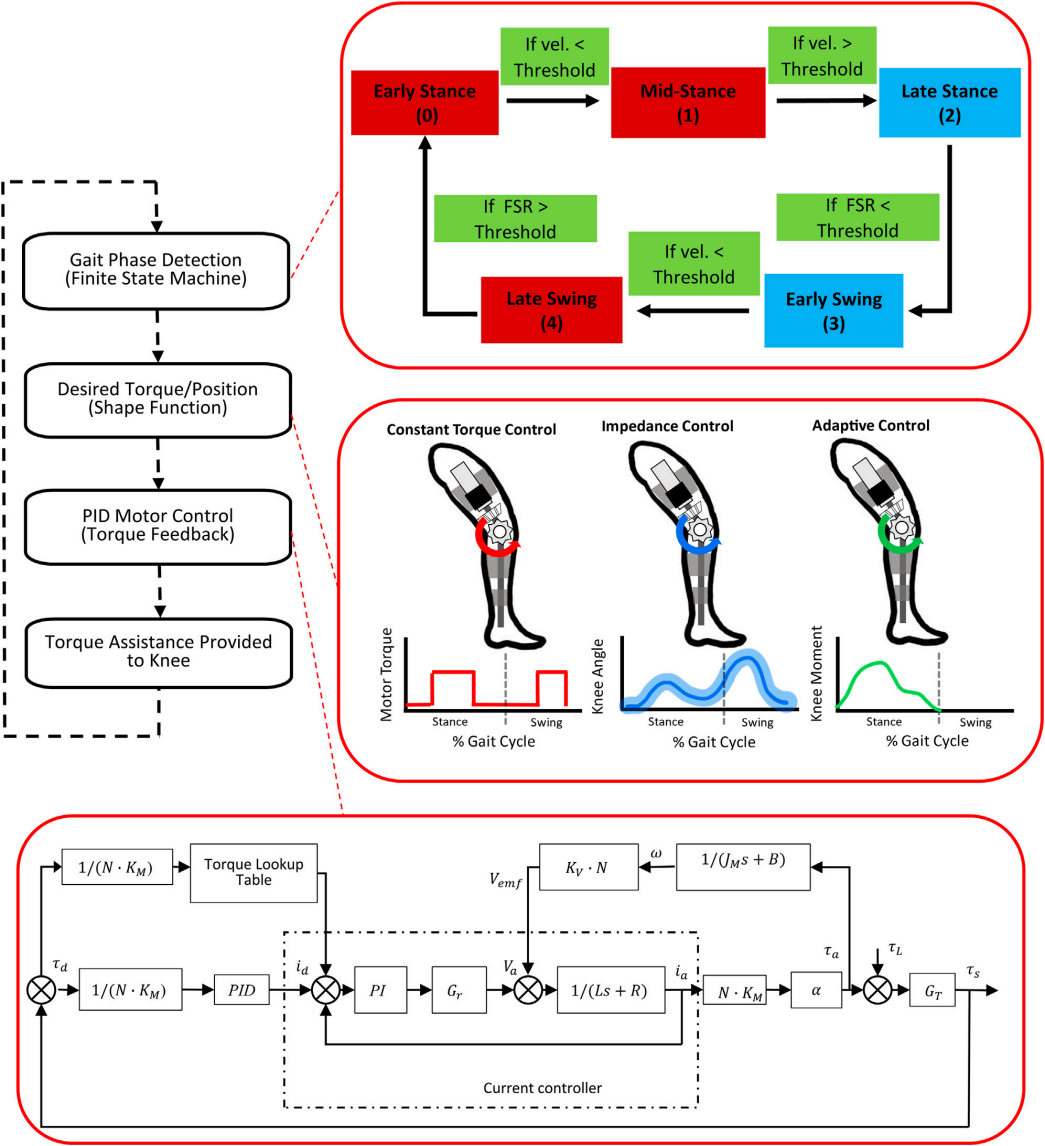
The hierarchical real-time control loop running on the embedded microcontroller during exoskeleton operation. The diagram on the top right inset shows the 5 state finite state machine. Red blocks are states where extension assistance was provided. The middle right inset shows the three different assistance modes of the P.REX. The diagram in the bottom right inset shows the feed-forward and feedback closed loop control for torque delivery within the selected mode.

The control architecture is designed to regulate knee extension assistance during stance and swing phases in real time (Figure 2). The hierarchical controller infrastructure is implemented in three levels. The highest or supervisory level consists of the finite state machine (FSM), which utilizes the FSRs and encoders to parse the gait cycle into discrete states or phases. The FSM is flexible in that it can split the gait cycle in four different ways: 1) 2 states: stance/swing, 2) 3 states: stance/early swing/late swing, 3) 4 states: early stance/late stance/early swing/late swing, and 4) 5 states: early stance/mid-stance/late stance/early swing/late swing.

Within each state, the controller can activate three different operational modes for providing assistance (Figure 2). The first is a pre-defined profile of assistive torque, including the ability to impose a constant level of assistance within each gait phase or state, as was reported previously (Lerner et al., 2016a). This mode, which is referred to in this manuscript as Constant mode, is available for all levels of the FSM (e.g., 2-, 3-, 4-, and 5-states). This mode also includes a frictionless operating condition in which a net zero torque (i.e., Zero mode) is imposed across the knee to compensate for inherent friction of the motor assembly and allow unencumbered knee motion.

The second mode is an impedance control mode in which a desired knee angle trajectory is specified and the assistive torque is proportional to the distance of the subject’s knee angle from the desired trajectory subject to a maximum torque limit or “virtual wall” (Brokaw et al., 2011). The desired trajectory is defined in the task space, which makes this mode independent of time, and thus gait speed depends on the user.

The third mode is an adaptive control mode which provides assistive or resistive torque proportional to the estimated internal knee moment. The goal of this mode is to enhance the volitional effort from the user during overground gait training. We previously developed element-wise knee joint moment estimation models during overground walking in children with CP (Lerner et al., 2016b; Chen et al., 2019). The knee joint of children with CP can be modeled as a dynamic spring during stance phase, with separate stiffness values assigned to the knee joint during flexion and extension. The current estimation model used data from the baseline walking (without exoskeleton) to estimate the stiffness values for each participant. This personalized model breaks the stance phase into 3 sub-phases (early, middle and late stance) and 3 transitions (peak knee flexion point, peak knee extension point, and toe-off point). Within each subphase, our estimation model calculated the instantaneous biological knee moment by using the product of the dynamic stiffness and the change in knee angle and moment estimation at the transitions. Knee moments at transitions are estimated by using linear predictive equations that include parameters such as subject weight, measured knee angle at heel strike and peak knee flexion and extension angle during stance from baseline working trials (Chen et al., 2019). Because the dynamic stiffness remains relatively constant for an individual, this approach enables a real-time estimate of biological knee moment during walking. This real-time approach was previously validated using offline data (Chen et al., 2019). The adaptive mode was only utilized with a 2-state FSM in the current study. Additionally, adaptive knee extension assistance was only active during stance phase because model predictions were limited to that phase; in swing phase the frictionless operating condition was activated.

The lowest level of control consists of the sensor and actuator layer to deliver the desired torque level from the motor (Chen et al., 2018) (Figure 2). Our design goal was to achieve short time delay in reaching the desired output torque and reduce un-modeled disturbances. Unknown disturbances and modeling errors can originate from either the motor itself or the external environment through the limbs. Here, we used a simple PID based feedback control scheme and combined it with feedforward compensation and a torque lookup table. The feedforward control enables the powered exoskeleton to reach the desired torque level faster than feedback control alone for the higher torque set points. The feedback control fine-tunes the torque to the required value. The torque lookup table was used to offset the discrepancy between input and output current inherent in the ESCON 50/8 current controller. The desired torque is mapped to a command current based on the gear ratio, motor torque constant and transmission efficiency. The command current was sent to ESCON 50/8 current controller to achieve the desired torque output. The PID error is computed as the difference between the measured and desired torque. A graphical user interface (GUI) was deployed to communicate/update the operational settings of the exoskeleton closed loop control system running on the embedded microcontroller and to receive data from the exoskeleton sensors and actuators during operation (Tucker et al., 2020).

### Device Benchtop Evaluation

We conducted benchtop tests similar to previous studies of torque-controlled actuators (Shepherd and Rouse, 2016; Kim et al., 2018) to characterize device performance in terms of response time, output impedance and bandwidth. Step response tests were performed by rigidly fixing the exoskeleton to an aluminum 80/20 extrusion frame. A 200 Hz sampling frequency without wireless communication was utilized to ensure accurate characterization of the PID controller dynamic response. Latency was defined as the mean time to reach 90% of the desired torque and was measured at three torque levels (5, 10, and 15 Nm). Output impedance was tested by perturbing the lower leg with a pseudo-sinusoidal displacement while desired torque was set to zero. Bandwidth tests were performed by applying desired torques as a 1–30 Hz exponential chirp signal, oscillating between −5 and 5 Nm. We used this exponential chirp to improve signal to noise ratio in the low frequency range (Kim et al., 2018). Desired and measured torques were transformed into the frequency domain using a Fast Fourier Transform and a Bode plot was generated using magnitude ratio and phase difference. The gain-limited and phase-limited bandwidths were calculated as the mean frequencies (over 5 trials) at which the amplitude ratio was −3 dB and the phase margin was 45 deg (Nise, 2015).

To estimate the P.REX maximum power, an aluminum bar was connected to the actuator shaft with a weight attached to the end of bar to exert 15 Nm gravitational torque about the shaft during its rotation. The swing velocity was calculated from the rotation angle recorded by the encoder.

### Experimental Evaluation

To evaluate the P.REX controller performance one volunteer with typical development (TD, male, 25 years old, 187 cm, 84.7 kg) and one participant with CP were recruited (male, 15 years old, 162 cm, 39.9 kg) for testing under our IRB approved protocol (#13-CC-0210). Informed consent was obtained prior to data collection. The data presented here from the participant with CP are part of a larger multi visit study evaluating the effects of robotic exoskeleton assistance in children with CP in which this participant is still active. Data collection consisted of overground walking trials under multiple conditions. All were performed at self-selected pace and consisted of at least 5 passes over a ∼6 m walkway.

The participant with TD completed one visit during which he walked under baseline condition without the exoskeleton and with the exoskeleton under the two different assistance modes (Constant and Adaptive) as well as the Zero mode. The orthotic components were not customized to this individual but were parts from a generic brace available in the lab. In the Constant mode, a 3-state (stance, early swing and late swing) and 5-state (early stance, mid-stance, late stance, early swing and late swing) FSM were evaluated with constant assistance torque set at 7 Nm during stance and late swing for 3-state FSM and during mid-stance and late swing for 5-state FSM. The adaptive control was tested with assistive torque set to 10, 20 and 30% percent of real-time estimated knee biological moment. The passive ankle joint of the exoskeleton was set to allow free rotation in all walking conditions.

The study design for individuals with CP allows up to a maximum of ten visits. The first visit is an assessment visit, including a medical history and physical, assessment of motor function via Gross Motor Function Classification System (GMFCS) and spasticity via the modified Ashworth scale (MAS). Virtual models of the participant’s legs were captured using 3D scanning technology (LifeNabled Digiscan 3D) for fabrication of custom thermoplastic thigh, shank, and foot shells for the P.REX exoskeleton. The second visit was for initial fitting of the exoskeleton and tuning of the exoskeleton control system, including the thresholds for state transitions of the FSM and the assistive torque levels. Here, Constant mode (5-state FSM) and Adaptive mode (2-state FSM) were chosen as the assistance modes for study. For the Constant modes, two different timings of assistance were used, one in which constant assistance was provided during early stance, mid-stance and late swing (Est+Mst+Lsw) and one in which assistance was provided during mid-stance and late swing (Mst+Lsw). In the Adaptive mode, assistance was provided during stance phase that was scaled at 40% of the estimated knee moment. These moments are estimated from our real-time knee moment estimation algorithm using parameters determined from baseline walking during the first visit (Chen et al., 2019). The passive ankle joint of the exoskeleton was adjusted to match the settings of the user’s AFO, which included limiting dorsiflexion to approximately 25 deg.

After the second visit, the CP participant entered the initial data collection and accommodation phase which included four additional visits (3–6). There was approximately one week between each visit. On the 7th visit kinematic motion capture data were collected during overground walking with the exoskeleton for gait analysis. The 5-state FSM walking condition (Est+Mst+Lsw) and the Adaptive mode of assistance were completed as described above along with a third baseline condition in which no exoskeleton assistance was provided (Zero). During all walking bouts, the participant used two forearm crutches for assistance, but otherwise walked independently. An overhead harness (ZeroG, Aretech, Ashburn, VA) was worn for safety in the event of a fall but did not provide body weight support.

### Data Analysis

Kinematic data were collected at 100 Hz using Vicon MX system with 12 T40 cameras (Vicon Motion Systems, Inc; Denver, CO) and two custom marker sets: one placed on the exoskeleton and one placed directly on the limbs so their movements could be measured independently (Lerner et al., 2016a). Prior to evaluating exoskeleton performance, compliance between the exoskeleton and the limb was evaluated as the difference between the sagittal plane knee joint angle computed from the exoskeleton markers and the biological knee angle computed from markers placed directly on the limb. The mean compliance over the gait cycle was 4.6 ± 3.2 deg for the participant with TD. Maximum compliance was observed during swing phase, when angular velocities were the highest. Ground reaction force data were collected at 1,000 Hz from 3 AMTI force plates (Advanced Medical Technology, Inc; Watertown, MA). Lower-extremity joint angles were computed from marker trajectories in Visual 3-D software (C-Motion, Gaithersburg, MD, United States) and all other analysis was performed in Matlab (Mathworks, Natick, MA). Walking data were segmented by gait cycle using successive heel-strikes, time normalized using cubic spline interpolation, and finally averaged across gait cycles for each walking condition. The sampling rate for exoskeleton sensor data was 30 Hz for the healthy participant and was later increased to 100 Hz for the participant with CP. At the beginning of each trial, a pulse was sent from the exoskeleton control units to the Vicon system to synchronize motion capture data (marker trajectories, ground rection forces, and EMG) with data from the exoskeleton sensors. In the TD participant, the primary purpose of the gait analysis was to validate the design of the various control modes to apply assistive knee extension torques in real-time during overground walking. We evaluated peak knee extension and gait speed for each exoskeleton mode. Given the relatively small assistance levels relative to the TD participant’s size and capabilities we did not anticipate a significant effect on gait. Exoskeleton control system performance validation was assessed by analysis of state transitions and applied torque magnitude compared with measured knee joint angle and foot-ground contact. To simplify presentation across the numerous conditions, data from this participant are shown for a single limb, with the noted observation that walking was symmetric. The total number of analyzed gait cycles for each condition is shown in Table 2.

**TABLE 2:** Spatiotemporal and kinematic outcomes during exoskeleton walking in participant with typical development^a^.

Exoskeleton condition	Baseline	Zero	3 state	5 state	Adaptive		
			7 Nm	7 Nm	10%	20%	30%
Number of gait cycles	13	11	7	9	12	11	14
Max knee flexion angle stance (deg)	11.2 (2.4)	10.3 (2.3)	8.9 (2.7)	8.5 (2.9)	7.2 (1.4)	7.1 (2.2)	6.6 (1.4)
Peak knee extension angle stance (deg)	1.7 (1.1)	2.1 (2.0)	0.2 (1.2)	−0.7 (0.6)	2.1 (1.7)	3.4 (1.3)	2.3 (1.3)
Knee range of motion (deg)	60.8 (0.9)	50.9 (3.8)	60.2 (1.8)^b^	63.5 (1.6)^b^	49.6 (1.9)	54.0 (2.8)	54.5 (2.2)
Step length (cm)	62.3 (2)	53.6 (3)	55.6 (4)	56.9 (2)	56.6 (2)	57.8 (2)	58.5 (3)
Step width (cm)	19.7 (2)	22.5 (3)	21.2 (2)	23.2 (2)	21.7 (3)	21.5 (1)	21.1 (2)
Cadence (steps/min)	113 (3)	98 (3)	103 (3)	100 (4)	99 (4)	102 (5)	103 (4)
Gait speed (m/s)	1.28	0.45	0.49	0.40	0.48	0.48	0.42

a Data are presented as mean (standard deviation).

b Significant difference with Zero condition.

For the participant with CP, the purpose of this study was to compare the effects of different exoskeleton assistance modes (Constant and Adaptive) on overground gait using peak knee extension during stance phase and gait speed as the primary outcome measures. Muscle activity was also collected from four primary muscle groups (rectus femoris, medial hamstring, vastus lateralis, and gastrocnemius) from both legs by using a wireless EMG system (Trigno, Delsys, Boston, MA) recorded at 1000 Hz. EMG data were band-pass filtered at 15–380 Hz, full-wave rectified, and low-pass filtered at 7 Hz to create a linear envelope (Lerner et al., 2017a). An initial gait analysis session was performed at the start of the 3rd visit for the Constant modes (Est+Mst+Lsw and Mst+Lsw). Those data were reported previously for this participant and showed that the best improvement in knee extension comes from the (Est+Mst+Lsw) Constant mode (Bulea et al., 2020). The data presented for this study are from the 7th visit, following 3 additional practice sessions with both the 5-state Constant mode (Est+Mst+Lsw) and the Adaptive mode providing extension assistance equal to 40% of the estimated instantaneous knee moment. Gait analysis was performed in three exoskeleton modes: Zero, which served as a control; Constant mode with 3 Nm of knee extension assistance provided during early stance, mid-stance and late swing (Est+Mst+Lsw); and Adaptive mode with knee extension assistance provided during stance phase at a magnitude of 40% of the instantaneously estimated knee extension moment. Owing to the single subject design, statistically significant differences between Zero mode and the exoskeleton assistance modes (Est+Mst+Lsw and Adaptive) were determined using the two standard deviation band method (Nourbakhsh and Ottenbacher, 1994). In addition to the primary outcomes evaluating differences across exoskeleton modes at the last data collection, changes in sagittal plane biomechanics at the hip, knee and ankle and changes in the EMG linear envelope were also assessed in the participant with CP.

## Results

### Exoskeleton Performance Characterization

**FIGURE 3:**
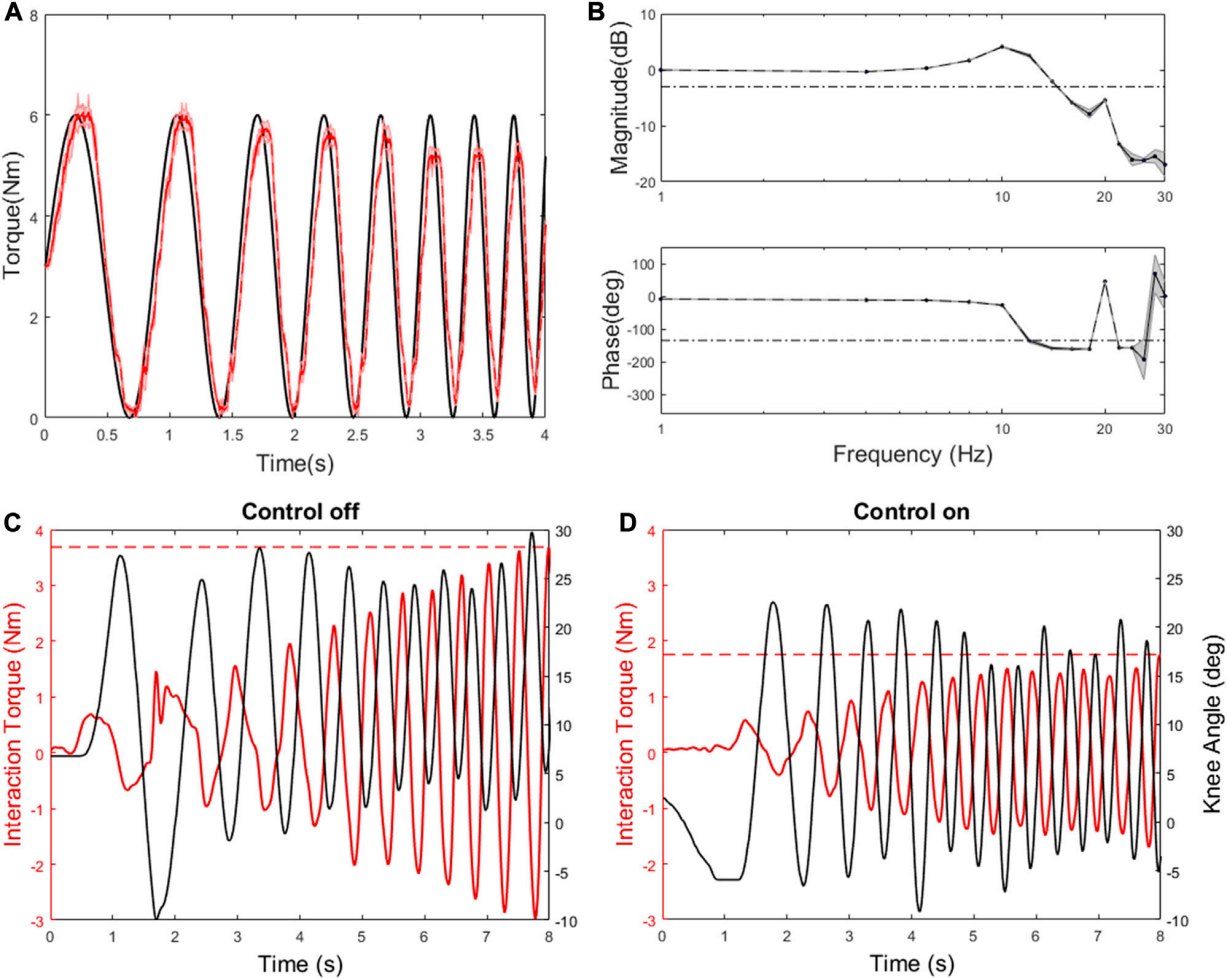
Characterization results: **(A)** An example of torque tracking: the torque was measured (red) as a desired torque was commanded as 1–5 Hz chirp (black). **(B)** Bode plot: the dashed lines represent −3 dB in the magnitude plot and 45 deg phase margin in the phase plot. **(C)** The output was manually back-driven with the control off. The black line indicates knee angle. The measured torque (red) is due to reflected inertia and damping in the transmission. The dashed red line indicates maximum interaction torque. **(D)** With the torque control on and a desired torque of zero (i.e., Zero mode), the output is back-driven more easily.

Step response, torque tracking of a chirp signal, torque bandwidth and output impedance test were performed for bench testing. The 90% rise and fall times between 0 and 5 Nm of assistance torque were 26 ± 0.2 ms and 45 ± 1.1 ms. The 90% rise and fall times between 0 and 10 Nm in assistance torque were 22 ± 0.2 ms and 47 ± 3.1 ms. The 90% rise and fall times between 0 and 15 Nm in assistance torque were 32 ± 0.4 ms and 41 ± 4.4 ms. The root mean square torque errors (RMSE) for the steady state across the five trials at each respective torque setting were 0.05, 0.13, and 0.43 Nm. With a desired torque oscillating between 0 and 6 Nm for flexion/extension the averaged −3 dB magnitude cross over frequency was at about 15 Hz and the 45 deg phase margin was present at about 12 Hz (Figures 3A,B). The interaction torque during the manual disturbances was reduced by half with control on (Zero mode; 1.76 Nm maximum) compared to control off (3.69 Nm maximum; Figures 3C,D). Thus, Zero mode results in low output impedance reducing unwanted interaction torque.

### Exoskeleton Validation in Participant With Typical Development

**FIGURE 4:**
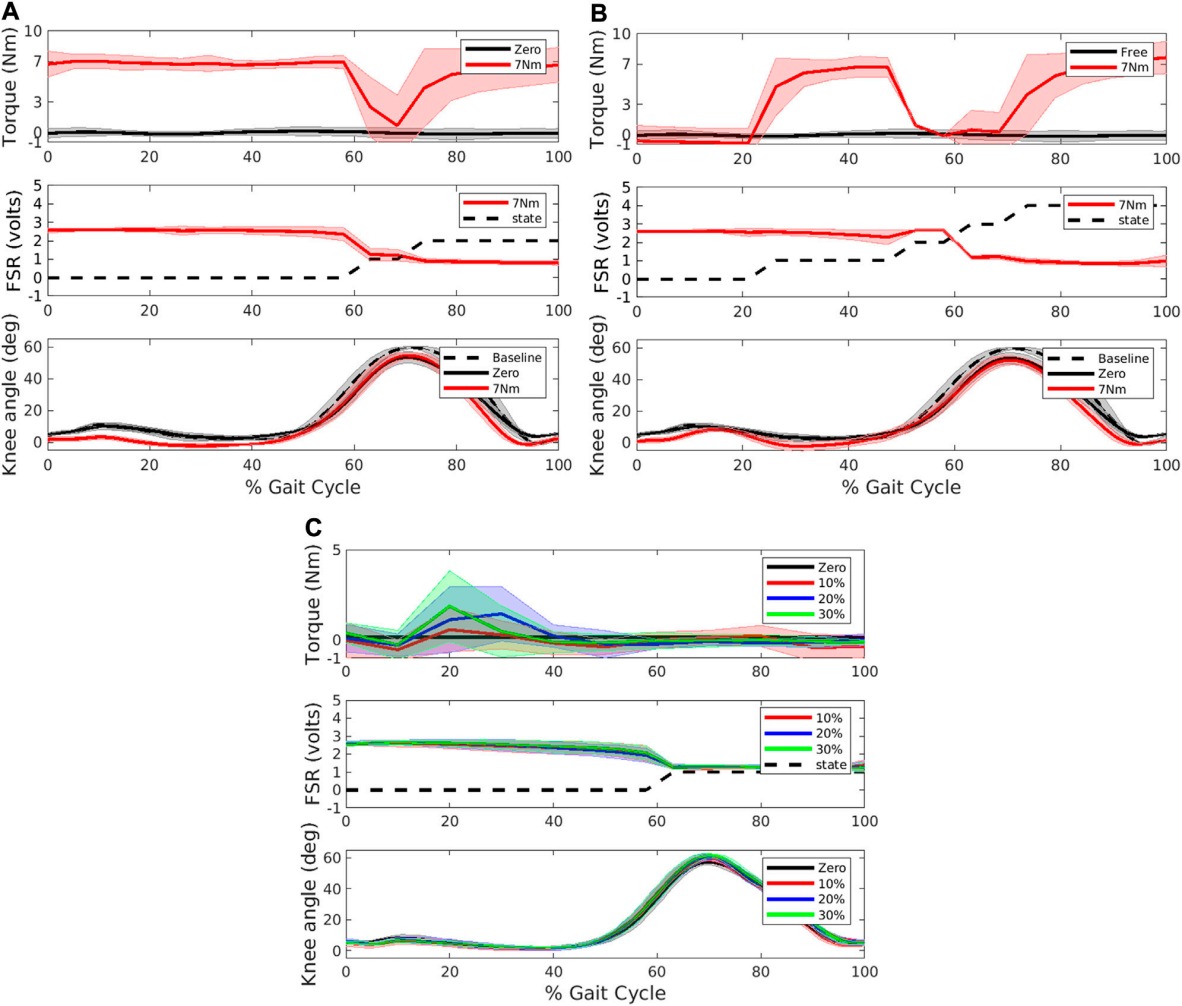
Torque (top panel), force sensitive resistor (FSR) and state number (middle panel) and knee angle (bottom panel) collected during the different exoskeleton modes during overground walking in a participant with typical development. Constant mode was evaluated with a **(A)** 3-state FSM and **(B)** 5-state FSM. In the torque and angle subplots, dashed black line represents baseline walking without the exoskeleton, black line represents Zero mode, and red line represents the respective Constant mode with 7 Nm assistance torque, respectively. In the FSR subplot, dashed black line represents state level at each point of gait cycle. For 3-state, level 0 represents stance phase, level 1 early swing, level 2 late swing. For 5-state, level 0 represents early stance phase, level 1 mid-stance, level 2 late stance, level 3 early swing, and level 4 late swing. **(C)** Adaptive condition. Black line represents Zero mode, red, blue and green lines represent extension assistance set at 10, 20, and 30 percent of estimated biological knee moment, respectively. In the FSR subplot, dashed black line represents state level (0 represents stance, and 1 is swing).

**FIGURE 5:**
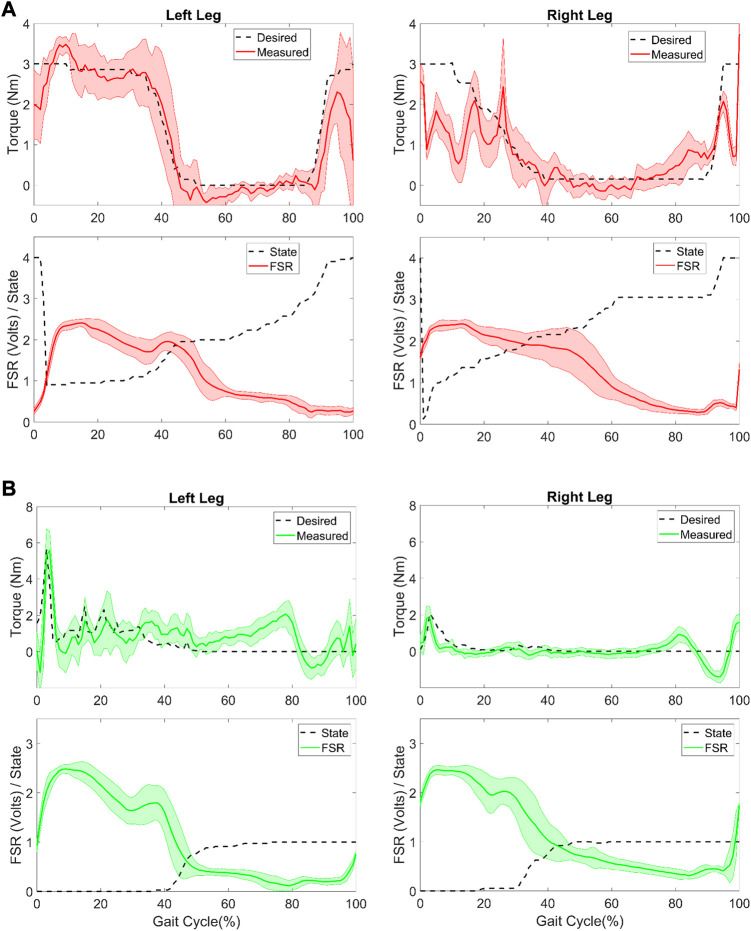
Controller evaluation during overground walking in the participant with CP. **(A)** Desired and measured torque (top row) for the Constant mode (Est+MSt+Lsw) controller and the (bottom row) measured force sensitive resistor (FSR) and FSM state (0: early stance, 1: mid-stance, 2: late stance, 3: early swing, 4: late swing) for the left and right leg. **(B)** Desired and measured torque (top row) for the Adaptive controller and the (bottom row) measured FSR and FSM state (0: stance, 1: swing) for the left and right leg.

The torque provided during walking with the 3-state (Figure 4A) and 5-state (Figure 4B) FSM Constant modes was consistent with design specifications. Specifically, assistive torque was maintained at the target level during stance and late swing in the 3-state condition while assistive torque was reduced to near zero in the early swing state. Similarly, in the 5-state mode assistive torques were confined to mid-stance and late swing phases during which the knee was extending. A FSR threshold was used to separate stance phase of gait cycle from swing phase. When the FSR reading from the foot sensor is greater than the FSR threshold this foot is determined to be in stance phase. A negative velocity threshold was used to identify the transition from early stance to middle stance and from early swing to late swing. A positive velocity threshold was used to identify the transition from mid-stance to late stance. All the transitions proceeded according to design. Compared to baseline, peak knee extension was not significantly different in the Zero mode (Figure 4). However, compared to Zero mode, peak knee extension during stance was slightly increased at the 7 Nm level in both the FSM 3-state condition and further increased at the FSM 5-state condition, although neither reached statistical significance. Compared to baseline, gait speed was significantly reduced in all assistive FSM walking conditions as shown in Table 2. There were no differences in gait speed across the Zero, Constant and Adaptive modes.

In the Adaptive mode, knee extension assistance was applied during stance phase proportional to the estimated instantaneous knee extension moment (Figure 4C). The torques provided during walking with the adaptive controller were in line with design specifications and were small. Compared to the Zero mode, the Adaptive control showed no significant differences in peak knee extension angle during stance in any of the three conditions. Compared to baseline mode, gait speed was significantly reduced in all three adaptive conditions as shown in Table 2 although there was no difference with the Zero mode.

### Comparison of Exoskeleton Assistance Modes in a Participant With CP

The participant with CP met all inclusion criteria for the study and was assessed as GMFCS Level III. For daily mobility over short distances he wore AFOs and used forearm crutches for assistance. The participant had mild spasticity in the right limb (MAS scores: rectus femors: 1+; hamstring: 1; vastus lateralis: 1, gastrocnemius: 1) and left limb (MAS scores: rectus femoris: 1; hamstring: 1+; vastus lateralis: 1; gastrocnemius: 1). The knee extension assistance was set at 3 Nm (0.075 Nm/kg) for both the left and right legs during the FSM constant torque condition (Est+Mst+Lsw). For the Adaptive control, assistance torque was provided only during stance phase and was set at 40% of estimated knee extension moment.

Gait was asymmetric between limbs. Although the constant mode torque setting was 3 Nm during Est+Mst+Lsw this was variable across strides as the participant’s walking varied by stride as shown in Figure 5A. For example, in some strides knee extension stopped relatively early (i.e., 15–25% of gait cycle), especially in the right leg causing a premature shift to late stance phase whereas in the left leg extension continued later into stance (i.e., 40–50% of gait cycle). Torque provided during late swing was more consistent stride-to-stride compared to torque provided during stance in the constant mode. The root mean square error (RMSE) between the desired and measured knee torque in the Est+Mst+Lsw mode was 0.49 and 0.81 Nm for the left and right leg, respectively. Overall, in the Adaptive mode the assistance shape was consistent with knee moment, that is, there was a large burst of assistance during early stance in both left and right limbs and very little otherwise. The assistance was asymmetric during Adaptive mode, in line with the gait pathology asymmetry. Thus, the assistance during the Adaptive mode, which was proportional to the estimated knee extensor moment, was greater for left knee compared to the less affected right knee (Figure 5B). RMSE between the desired and measured assistive torque in the Adaptive mode was 0.89 and 0.35 Nm for the left and right leg, respectively.

**FIGURE 6:**
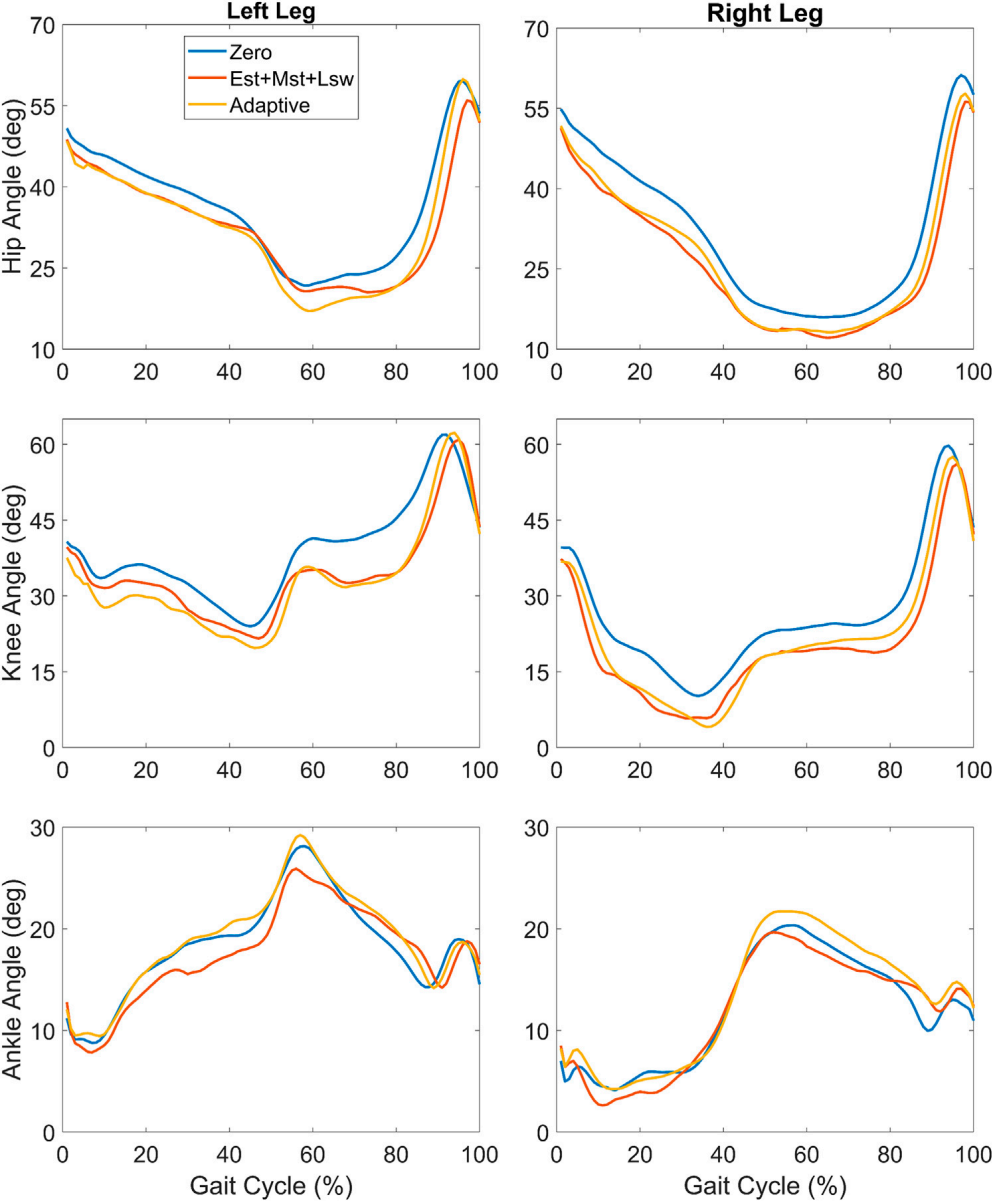
Mean lower extremity joint angles across the gait cycle during overground walking with exoskeleton assistance provided by the Constant mode during early stance, mid-stance and late swing (Est+Mst+Lsw; orange), Adaptive mode (yellow) and a baseline condition with no extension assistance (Zero; blue).

There was a significant effect of robotic assistance on lower extremity kinematics during overground walking (Figure 6; Table 3). The most apparent difference in kinematics was observed in the knee joint of both limbs which each showed a shift toward greater knee extension during both the Est+Mst+Lsw and the Adaptive exoskeleton walking conditions compared to Zero mode. The largest change in knee angle occurred during the midstance phase (∼20–50% of the gait cycle in the left limb; ∼15–40% of the gait cycle in the right limb; Figure 6). In both limbs, the hip was slightly more extended during the stance phase of both assistance conditions compared to Zero mode. The ankle profiles were similar between exoskeleton walking with and without knee extension assistance, except for the mid and late stance phase (20–65%) on the left limb and the mid-stance phase (10–20% gait cycle) on the right limb which showed slightly reduced dorsiflexion in the constant torque assistance condition (Figure 6).

**TABLE 3:** Spatiotemporal and knee angle outcomes during exoskeleton walking in participant with CP.

Condition	Zero		Est+Mst+LSw		Adaptive	
	Left	Right	Left	Right	Left	Right
Number of gait cycles	16		19		17	
Gait speed^a^ (m/s)	0.23 (0.02)		0.17^b^ (0.02)		0.21 (0.03)	
Step length (m)	0.28 (0.03)	0.43 (0.03)	0.29 (0.04)	0.37^b^ (0.05)	0.29 (0.03)	0.41 (0.04)
Peak knee angle (deg)	25.9 (2.5)	13.1 (7.1)	18.9^b^ (4.1)	2.9 (5.5)	16.9^b^ (4.1)	2.0^b^ (2.4)

a Data are presented as mean (standard deviation).

b Significant difference with Zero condition.

In the less crouched right limb, peak knee extension was markedly greater than the Zero mode during both Est+Mst+Lsw and Adaptive modes although only the Adaptive mode reached statistical significance (Table 3). Similarly, in the left limb mean peak knee angle was the most extended in the Adaptive mode though both Est+Mst+Lsw and Adaptive modes showed a significant increase in peak knee extension compared to Zero mode (Table 3). Overground gait speed was slow ranging from 0.17–0.23 m/s across the three exoskeleton conditions (Table 3). Gait speed was significantly reduced in the Est+Mst+Lsw condition compared to Zero mode whereas there was no significant difference between gait speed in the Adaptive mode compared to Zero (Table 3). Step length was asymmetric and greater for the right leg than the left for all exoskeleton modes. There were no significant differences in step length across the three exoskeleton conditions on the left leg, however step length was significantly reduced in the right leg for the Est+Mst+Lsw but not for the Adaptive condition (Table 3).

**FIGURE 7:**
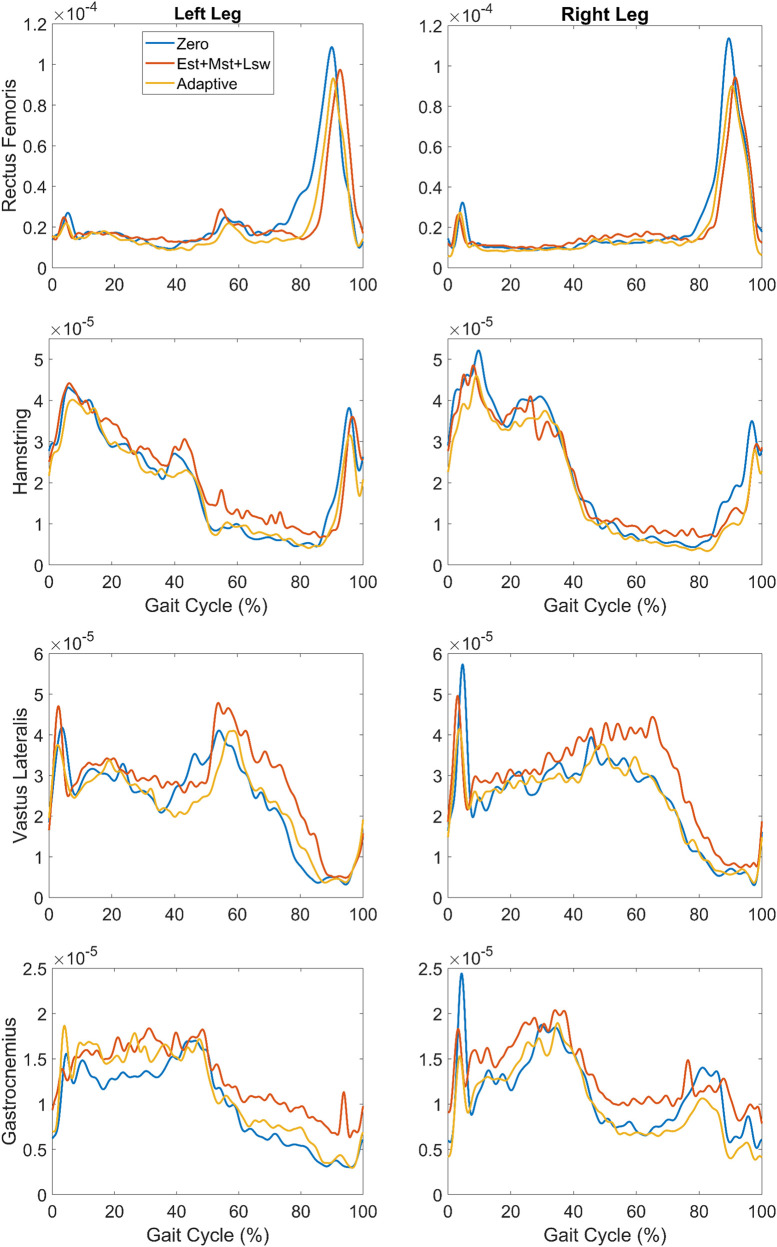
Mean EMG linear envelopes for rectus femoris, medial hamstring, vastus lateralis and gastrocnemius plotted vs. percent gait cycle during overground walking with exoskeleton assistance provided during Est+Mst+Lsw (orange) and Adaptive (yellow) modes, as well as Zero mode with no extension assistance (blue).

Overall, EMG data showed minimal differences across the three exoskeleton walking modes (Figure 7). Interestingly, hamstring EMG was the same or only slightly lower in Adaptive mode compared to the Zero mode, especially in early stance phase, despite the difference in torque profile. This suggests the assistance was well tolerated and did not exacerbate any spastic responses. Vastus Lateralis activity was slightly lower in the Adaptive mode compared to Est+Mst+Lsw, especially in mid and late stance, as well as swing phase. This could be due to the slightly increased extension posture of the limb. Gastrocnemius activity was lower in Adaptive mode compared to Est+Mst+Lsw on the right limb during nearly the whole gait cycle, and on the left limb during the swing phase.

## Discussion

Here we describe the mechanical and electrical design of a pediatric exoskeleton to support long term studies providing multiple control approaches to augment knee extension using robotic assistance in children with active knee extension deficiency, such as crouch gait from CP. The primary purpose of the experiment with the typically developing participant was to examine the exoskeleton performance during overground walking. Secondarily, the effects of the different control approaches on gait biomechanics of the user were examined, although we did not expect large if any changes because the participant was not neurologically impaired. Overall, the two exoskeleton assistance modes (Constant and Adaptive) performed as specified. The effect of robotic knee extension assistance was assessed by comparing these two assistive modes to Zero mode that compensates for the effect of exoskeleton weight and inertial properties on biomechanics. The effect of exoskeleton inertia and robotic extension assistance was small as there was no significant difference in range of motion between when the participant with TD walked in Constant mode and baseline. The peak knee extension slightly increased during stance phase (Table 2). This was expected given that the participant was healthy and the assistance to body weight ratio was small. In the Adaptive mode, the knee range of motion slightly decreased compared to baseline and Constant mode. As the assistance levels increased from 10 to 30%, the peak knee extension, the range of motion, step length, and cadence shows an increasing trend. The gait speed among all three levels is also comparable to Zero mode and all FSM conditions, however, is much less than the baseline condition. That gait speed was significantly reduced in all exoskeleton conditions in the healthy participant, including the Zero mode, indicates that walking with the exoskeleton is disrupting compared to walking without it and is in line with our previous findings in children with CP (Lerner et al., 2017a). Nevertheless, the Adaptive mode aimed to provide a more responsive and intuitive mode of assistance. The data from the participant with typical development in this mode has provided evidence to support this aim. The same amount of knee extension increase was observed with the least overall assistance torque.

Here, the analysis of the participant with CP focused on comparison of a constant mode of assistance: FSM 5-state (Est+Mst+Lsw) mode and the Adaptive mode providing assistance equal to 40% of the estimated knee extension moment. The Adaptive mode used our real-time knee moment estimation (Chen et al., 2019) based on the knee moment data collected from baseline walking conditions. The estimation is limited to the stance phase as the model-based prediction algorithm requires parameters (i.e., dynamic stiffness values) extracted from the measured knee moment in baseline walking derived from force plate data. To our knowledge, this is the first time that a real-time knee moment estimation has been evaluated when used to provide extension assistance in a child with crouch gait from CP.

Providing extension assistance in Adaptive mode resulted in improvements in knee extension that were slightly but not significantly greater than Est+Mst+Lsw mode on average in both limbs (Figure 6; Table 3). Interestingly, this result was observed despite lower magnitudes of assistive torque delivered for a shorter duration of the gait cycle (Figure 5) compared to the Est+Mst+Lsw mode. The likely explanation is that the Adaptive mode provided knee extension assistance that was synergistic with the volitional knee extension moment during stance phase of walking (Figure 5B) resulting in greater benefit in posture. As was observed in the constant (Est+Mst+Lsw) mode, extension assistance in the Adaptive mode also improved extension at the hip.

There was a marked reduction in gait speed (Table 3) for all exoskeleton modes (Zero, Est+Mst+Lsw, and Adaptive) compared to the user’s speed when not using the exoskeleton (0.32 m/s). Interestingly, gait speed during Adaptive mode was not significantly different from walking in the Zero mode, while speed was significantly lower in the Est+Mst+Lsw (Table 3), suggesting that Adaptive mode may be less disruptive to walking speed. In our prior study in children with crouch who were GMFCS I/II gait speed was reduced during the first data collection session but after 3 practice sessions gait speed returned to baseline levels (Lerner et al., 2017a). In this individual who was GMFCS III gait speed remained below baseline suggesting that more affected individuals may take longer to accommodate to exoskeleton assistance. Step length was observed to be asymmetric in this participant. Left step length showed no statistical difference among the two assistance and Zero modes. However, in the right leg only the Adaptive mode showed no difference in step length with Zero mode whereas the Est+Mst+Lsw mode showed a slight but significant reduction. Taken together, the results suggest that for this participant, the Adaptive mode may provide the best improvement in walking as knee extension improvements were comparable with the higher duty cycle assistance of Est+Mst+Lsw without the deleterious effects on gait speed and step length. Additionally, the participant reported that he felt most comfortable using the Adaptive mode when queried at the end of the data collection session, even though he was blinded to how the controller was functioning during the different trials, further suggesting that adaptive control may be more intuitive than providing constant state-based assistance.

The bilateral EMG recoding showed that the hamstring, vastus lateralis and gastrocnemius activation in Adaptive mode shared a similar pattern with the Zero mode. Also, the vastus lateralis and gastrocnemius activations were reduced compared to the same muscle groups in Est+Mst+Lsw mode. This reduced muscle activation may be beneficial in reducing the effort necessary for walking after long term training. However, all kinematic and EMG results were only obtained in a single CP participant. A larger study is ongoing to compare the effects of Adaptive mode vs. Constant mode in children with CP with a range of functional levels (GMFCS I-III).

In theory, robotic exoskeletons provide multiple potential benefits for gait rehabilitation, including training intensity and active participation by the user during training. It has been found that these two elements improve neurorehabilitation efficiency, especially when the level of assistance provided by exoskeletons can be adapted to the user’s motor capacities (Burdet et al., 2013), providing additional support for the larger goal that a neurorobotic device should facilitate the user’s movement without suppressing motor capability (Hogan and Krebs, 2004). To achieve this aim, controller development for lower limb exoskeletons should continue to focus on user specific adaptations. However major challenges with designing an adaptive controller are the user dependent response to device assistance, the dynamic pattern of motor learning as well as the complexity of neuromusculoskeletal system. A few control strategies have been developed to address these challenges. Our adaptive approach models the knee joint as a dynamic spring during stance with separate stiffness values for flexion and extension determined from baseline walking data (Chen et al., 2019). Based on the preliminary findings here, this adaptive control appears to be both easy to implement and intuitive while also providing similar benefit as the state-based constant control. Unlike human-in-the-loop strategies (Jackson and Collins, 2019) or other strategies using EMG (Kwon et al., 2012), our method provides a straightforward way to estimate volitional intention during walking without the need for heuristic-based algorithms or assumed relationships between biomechanics or muscle activity and movement intention, which can be disrupted in children with movement disorders such as CP. In the future, our adaptive control strategy may be improved by adding automatic updates of the stiffness values using knee moments estimated from prior gait cycles while walking with the exoskeleton and by extending the adaptive model to include the swing phase. Another approach is to ensure accurate tracking of the knee joint desired trajectory while allowing limited errors (Sherwani et al., 2020). Although not evaluated here, we have implemented a knee trajectory-based strategy in P.REX (impedance mode). A future strategy may involve developing a sequence of trajectories which progressively deviate from the participant’s baseline knee trajectory toward the desired (i.e., less crouched) trajectory, thereby gradually progressing toward walking in a less crouched posture. We plan to test this mode with the CP participants in the future as well.

## Conclusion

In summary, our results validate two exoskeleton assistance modes (Constant and Adaptive) during overground walking in an adult without gait pathology. The ability to provide different control strategies for assistance in the same hardware is innovative and will enable personalization of robotic assistance to children with CP without any necessary changes in hardware, electronics or software. The results from a single participant with CP demonstrate the utility of having the ability to provide different strategies through an example workflow. The child can walk in a clinical facility with each of the modes (or a subset of them) and determine which one results in the best improvement in function and/or has the best potential for improving function after longer term training. In this case it was the Adaptive mode which showed the best improvement in peak knee angle with the least effect on gait speed and no unwanted EMG effects (i.e., no increased spastic response to extension assistance). The next step is to continue training with the Adaptive mode to determine if improvements accrue over time and eventually while walking without the exoskeleton. The results shown here are preliminary and focused only on one individual with CP but there are sufficient supporting data from this study to continue our ongoing investigation of personalized control strategies. We anticipate, based on the heterogeneity of CP, that the best method for providing knee extension assistance to improve crouch will vary by individual. We also plan to address design limitations in the form factor and size of the exoskeleton to improve robustness and support eventual studies outside the laboratory setting.

## Data Availability

The original contributions presented in the study are included in the article. Further inquiries can be directed to the corresponding author.
